# Profibrotic effects of angiotensin II and transforming growth factor beta on feline kidney epithelial cells

**DOI:** 10.1177/1098612X18805862

**Published:** 2018-10-22

**Authors:** Cyrina D van Beusekom, Tanja M Zimmering

**Affiliations:** 1 Veterinary Pharmacology, Pharmacotherapy and Toxicology, Institute for Risk Assessment Sciences, Faculty of Veterinary Medicine, Utrecht University, Utrecht, The Netherlands; 2 Boehringer Ingelheim Animal Health GmbH, Ingelheim, Germany

**Keywords:** Angiotensin II, TGF-β, renal failure, chronic kidney disease, fibrosis

## Abstract

**Objectives:**

The aim of this study was to evaluate the role of angiotensin II (AT-II) and its main mediator, transforming growth factor beta 1 (TGF-β1), in the development of feline renal fibrosis.

**Methods:**

Expression of marker genes indicating epithelial-to-mesenchymal transition (EMT), profibrotic mediators and matricellular proteins was measured in feline kidney epithelial cells (Crandell Rees feline kidney [CRFK] cells) after incubation with AT-II and/or TGF-β1.

**Results:**

Cells incubated with TGF-β1 or the combination of TGF-β1 with AT-II showed clear EMT with more stretched fibroblastic cells, whereas the cells incubated without TGF-β1 and AT-II (control) showed more epithelial cells. Gene expression of collagen type I (*COL1*), tenascin-C (*TNC*), trombospondin-1 (*TSP-1*), connective tissue growth factor (*CTGF*) and alpha-smooth muscle actin (*α-SMA*) increased significantly after incubation of the CRFK cells with TGF-β1 or TGF-β1 in combination with AT-II for 12 h. As incubation of the CRFK cells with only AT-II did not show any significant rise in gene expression of the above-mentioned genes, this was further investigated. In contrast to healthy feline kidney tissue, CRFK cells showed almost no expression of the AT-II type 1 (AT_1_) receptor.

**Conclusions and relevance:**

TGF-β1 significantly induced expression of the EMT marker gene α-SMA, profibrotic mediator *CTGF*, and fibrogenic proteins *COL1*, *TNC* and *TSP-1* in CRFK cells. The effect of TGF-β1 on myofibroblast formation was also observed by the stretched appearance of the CRFK cells. As CRFK cells expressed almost no AT_1_ receptors, this cell line proved not suitable for testing the efficacy of drugs that interact with the AT_1_ receptor. As AT-II stimulates the effects of TGF-β1 in mammals, the results of this study suggest an indirect profibrotic effect of AT-II besides the demonstrated profibrotic effect of TGF-β1 and thus the development of feline renal fibrosis. Modulation of EMT or proliferation of myofibroblasts could serve as a diagnostic tool and a novel therapeutic target to inhibit renal fibrogenesis, and could possibly serve in the therapy of feline renal fibrosis.

## Introduction

Chronic kidney disease (CKD) is one of the most common progressive diseases in older cats and the renin–angiotensin–aldosterone system (RAAS) is known to play a key role in the progression of the disease. The RAAS is upregulated early in CKD^1^ and plasma renin, aldosterone and angiotensin I and II have been demonstrated to be increased in the circulation of cats with experimentally induced CKD.^2^ The RAAS is responsible for progressive renal injury not only by increasing glomerular pressure, but also by direct fibroproliferative effects via the induction of a variety of pro-inflammatory and profibrotic mediators.^3^ Chronic RAAS activation contributes to further loss of nephrons via mechanisms such as vasoconstriction, glomerular hypertension, proteinuria and fibrosis.^4^

The inevitable consequence of CKD is renal fibrosis, a process that has been studied thoroughly in human medicine, but has still not been completely elucidated because of its complexity. Currently, four major protagonists have been suggested to be involved in CKD progression: myofibroblasts, epithelial cells, endothelial cells and immune cells.^5^ The origin of myofibroblasts varies, as it was found that these cells could be derived from resident interstitial fibroblasts, bone marrow-derived fibroblasts, tubular epithelial cells, endothelial cells and pericytes. The process in which epithelial cells convert to mesenchymal fibroblasts is called epithelial-to-mesenchymal transition (EMT). Although the number of fibroblasts formed by this process is small,^6^ EMT is responsible for more than just a morphological change of the tubular epithelial cells. EMT induces and inhibits expression of proteins involved in the function of tubular epithelial cells and impairs the repair of damaged tissue by inducing cell cycle arrest,^5^ eventually leading to renal fibrosis. Regardless of the aetiology, renal tubulointerstitial fibrosis is recognised as the pathological lesion best correlated with renal function in both humans and cats.^3,7–10^

**Figure 1:**
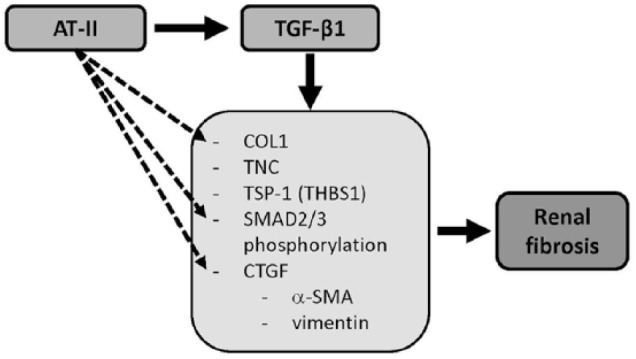
Schematic overview of the stimulating effects of angiotensin II (AT-II) and transforming growth factor beta 1 (TGF-β1) on gene expression leading to renal fibrosis. *COL1* = collagen type I; *TNC* = tenascin-C; *TSP-1* = trombospondin-1; *CTGF* = connective tissue growth factor; α*-SMA* = α-smooth muscle actin

It is known that angiotensin II (AT-II) is the main effector of RAAS and an important mediator in this progressive renal failure process. AT-II binding on the angiotensin II type 1 (AT_1_) receptor results in glomerular hypertension, which can promote further glomerular damage, proteinuria and activation of pro-inflammatory and profibrotic pathways.^1,11,12^ Transforming growth factor beta 1 (TGF-β1) has been described as the mediator that plays the main role in the developing process of renal fibrosis. AT-II contributes to renal fibrosis through TGF-β1 gene induction, an increased release of TGF-β1 and via induction of receptors for TGF-β1.^13^ TGF-β1 is responsible for the activation and proliferation of myofibroblasts and progression of renal fibrosis, as it induces the synthesis of the matrix proteins collagen type I (*COL1*) and tenascin-C (*TNC*)^14,15^ and mRNA expression of trombospondin-1 (*TSP-1*, also known as *THBS1*)^15^ in human kidney tubule cells. A schematic overview is given in Figure 1.

Renal myofibroblasts, which express alpha-smooth muscle actin (*α-SMA*), are considered as the principal matrix-producing effector cells that are responsible for fibrogenesis.^6,16^ TGF-β1 induces EMT of kidney cells, as measured by an increased expression of *α-SMA* and vimentin via induced gene expression of profibrogenic connective tissue growth factor (*CTGF*)^17^ and via SMAD2/3 phosphorylation.^15^ *CTGF* is not expressed in healthy human kidneys, but its level of expression has been shown to correlate with the severity and progression of renal fibrosis.^18^ AT-II also stimulates renal fibrosis independent from the actions of TGF-β1, via multiple kinases.^13^ Via activation of the AT_1_ receptor, AT-II induces the gene expression of *CTGF* and *COL1* in kidney tubule epithelial cells and human renal fibroblasts,^19,20^ and induces SMAD2/3 phosphorylation,^20^ subsequently leading to renal fibrosis and disease progression.

Calling a halt to progression of CKD in human and veterinary patients has been an interesting topic for quite some years now.^21^ Angiotensin-converting enzyme (ACE) inhibitors, such as benazepril, have, for years, been used as a treatment option for reducing proteinuria associated with CKD. In recent years the selective AT_1_ receptor blocker telmisartan has been an available alternative for reducing proteinuria in feline CKD patients. As the induction and proliferation of myofibroblasts has been correlated with the degree of disease in human and feline CKD,^6,9^ modulation of EMT or myofibroblast formation might offer a novel therapeutic target to inhibit renal fibrogenesis and could possibly also serve in the therapy of feline renal fibrosis.

The aim of this study was to gain mechanistic insights into the role of AT-II and TGF-β1 in feline renal fibrosis by measuring the expression of EMT marker genes, profibrotic mediators and proteins indicating renal fibrosis. A feline kidney epithelial cell line (Crandell Rees feline kidney [CRFK]) was used as an in vitro model for this study. CRFK cells have previously been used to study the effects of viruses on feline cells, but, to our knowledge, they have never been used to study the mechanisms behind feline renal pathology and to test drugs, which could have antifibrotic effects.

## Materials and methods

### Chemicals and reagents

AT-II was purchased from Sigma Aldrich and TGF-β1 was obtained from R&D Systems. Fetal bovine serum (FBS) was purchased from Invitrogen/ThermoFisher Scientific. Penicillin/streptomycin, DMEM and glutamine were obtained from Lonza. The feline kidney epithelial cell line (CRFK) was obtained from the European Collection of Cell Cultures, originally from American Type Culture Collection (LGC Standards).

### Cell culture

The CRFK cells were routinely passaged twice a week and cultured in DMEM supplemented with 10% FBS, penicillin (100 U/ml)/streptomycin (100 μg/ml), 2 mM glutamine and 1% (v/v) non-essential amino acids at 37°C in a humidified 5% CO_2_ atmosphere, until 80% of confluency was reached.

Cells were seeded in six-well plates at a density of 1 × 10^5^ cells/cm^2^ in 2 ml cell culture medium supplemented with serum. After culturing for 24 h, cells were washed once with PBS and incubated with serum-free medium for another 24 h. Hereafter, cells were incubated with either AT-II, TGF-β1 or a combination of AT-II with TGF-β1, all with an end concentration of 0.1% dimethyl sulfoxide. To determine which concentrations of AT-II and TGF-β1 could be used, a range of different concentrations of AT-II and TGF-β1 was tested, based on the literature.^14,15,17,20,22–24^ Stimulation of these cells with TGF-β1 or AT-II was all performed in the absence of serum and other supplements. Cells were collected in 250 µl cold RNA lysis buffer from the SV Total RNA Isolation System (Promega) and stored at −80°C until further processing. Samples were collected at 0 h, 6 h, 12 h and 24 h.

### RNA isolation, cDNA synthesis and quantitative RT-PCR analyses

#### CRFK cells

RNA was isolated from the CRFK cells by a spin column purification technique (SV Total RNA Isolation System; Promega). Aliquots of the purified RNA were measured spectrophotometrically and the RNA was stored at −80°C. RNA inclusion criteria were based on the ratio of absorbance at 260 nm and 280 nm (>1.8). For quantitative RT-PCR analysis, cDNA was synthesised using the iScript cDNA Synthesis Kit (Bio-Rad) according to the manufacturer’s instructions, using 1 µg feline RNA. Specific primers for *COL1*, *TNC*, *TSP-1*, *CTGF*, *α-SMA*, AT_1_ receptor and TGF-β receptor were developed based on highly conserved regions between human, feline and other animal gene sequences. Primers for the house-keeping genes were based on earlier studies,^25^ and stability of these primers were tested in the CRFK cells. Primer sequences are given in Table 1 and were produced by Eurogentec SA Belgium.

**Table 1:** Sequences of designed primers for quantitative RT-PCR analyses, with their optimal annealing temperatures

Gene	Forward primer sequence (5′→ 3′)	Reverse primer sequence (5′→ 3′)	Optimal annealing temperature (°C)
COL1	CTG-AAG-GCT-CTA-GGA-AGA-AC	CAT-AGT-GCA-TCC-TTG-GTT-AG	62
*TNC*	ACG-AAC-TGC-CCA-CAT-CTC-AG	TGA-TGG-TTT-GGG-TCC-GGA-TG	62
*TSP-1* (*THBS1*)	AGC-ATC-CGC-AAA-GTG-ACT-GA	CTC-CGT-TGT-GGT-AGC-AGA-G	59
*CTGF*	TTA-CCA-ATG-ACA-ACG-CCT-TCT-G	TTT-GCC-CTT-CTT-AAT-GTT-CTC-TTC	62
α*-SMA* (*ACTA2*)	GCA-TGG-GAC-AAA-AGG-ACA-G	TGG-TGA-TGA-TGC-CGT-GTT-C	59
*TGF-*β *receptor*	CTT-TTG-CCA-GGG-TTA-TCA-GTC-TCT	TCA-TTC-TTT-GTT-CTT-GCC-CAT-TC	62
*AT1R* (*AT_1_-receptor*)	AGC-CGG-CTC-CTG-TTC-TGT	TTC-CTG-TCG-CTC-CTC-TCA-AG	59
*RPS7* (housekeeping gene)	GTC-CCA-GAA-GCC-GCA-CTT-TGA-C	CTC-TTG-CCC-ACA-ATC-TCG-CTC-G	60

*COL1* = collagen type I; *TNC* = tenascin-C; *TSP-1* = trombospondin-1; *CTGF* = connective tissue growth factor; α*-SMA* = alpha-smooth muscle actin; TGF-β = transforming growth factor beta

SYBR Green technology was applied for the quantitative RT-PCR (qRT-PCR) analysis by using iQ Sybr Green Supermix (Bio-Rad), conducted according to the manufacturer’s instructions. The reaction was performed with a CFX PCR system (Bio-Rad) and analysed using CFX manager, Version 3.0 (Bio-Rad). After an initial hot start at 95°C for 3 mins, qRT-PCR was performed at 95°C for 15 s and 55–65°C for 45 s, for a total of 40 cycles. PCR products were subjected to melt curve analysis, demonstrating the formation of only one product.

#### Feline kidney tissue

Kidney tissue was obtained from four adult healthy European Shorthair cats (four males, aged ± 1 year) directly after euthanasia and samples were quickly frozen in liquid nitrogen and stored at −80°C. The cats had served as controls in other authorised studies and the animals were euthanased with the permission of the Animal Ethical Committee (DEC Utrecht DEC201518, reference number 0307.0601) and according to the Dutch law on Animal Experiments. RNA was isolated from 60–100 mg frozen kidney tissue of each cat and cDNA was synthesised as described for the CRFK cells. Samples were pooled afterwards for qRT-PCR analyses.

SYBR Green technology was applied for the qRT-PCR analysis by using iQ Sybr Green Supermix (Bio-Rad), conducted according to the manufacturer’s instructions. The reaction was performed with a CFX PCR system (Bio-Rad) and analysed using CFX manager, Version 3.0 (Bio-Rad). Gene expression of the AT_1_ receptor was determined with the forward primer 5‘-AGC-CGG-CTC-CTG-TTC-TGT-3’ and reverse primer 5‘-TTC-CTG-TCG-CTC-CTC-TCA-AG-3’. After an initial hot start at 95°C for 3 mins, qRT-PCR was performed at 95°C for 15 s and 59°C for 45 s, for a total of 40 cycles. PCR products were subjected to melt curve analysis, demonstrating the formation of only one product.

### Immunofluorescence assay

The AT_1_ receptor (*AT1R*) amino acid sequence was compared between humans, rats and cats. While the C-terminus of the protein seemed not to be well conserved between these species, the N-terminus was well conserved and could be used for immunofluorescent staining. For the CRFK cells and the healthy feline kidney tissue, the monoclonal rabbit antibody against human *AT1R* (Abcam) was used, as well as a secondary donkey anti-rabbit antibody (Jackson Immuno Research).

### Statistical analyses

Data were analysed by ANOVA followed by the Bonferroni post-hoc test (GraphPad Prism 6.05 software). Results were considered to be statistically significant when *P* <0.05.

## Results

### Cell culture and morphology

**Figure 2:**
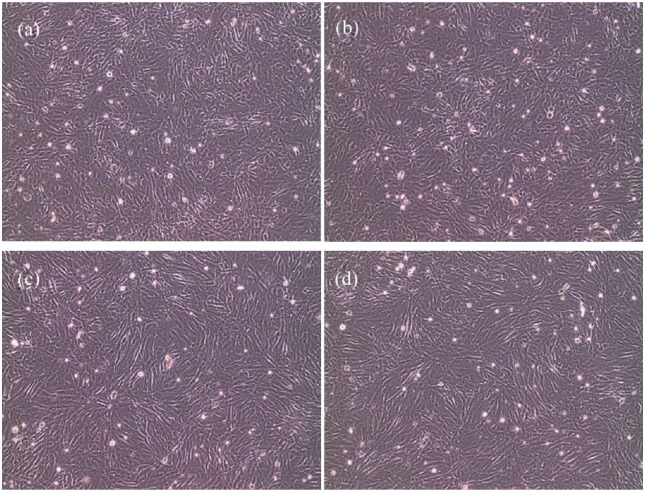
Crandell Rees feline kidney cells (× 10 magnification) incubated for 24 h with (a) control; (b) 1 × 10^-6^ M angiotensin II; (c) 2.5 ng/ml transforming growth factor beta 1 (TGF-β1); (d) 2.5 ng/ml TGF-β1 and 1 × 10^-6^ M angiotensin II

The optimal culturing conditions were found at a density of 1 × 10^5^ cells/cm^2^ in six-well plates with 2 ml cell culture medium supplemented with serum. It was concluded that 1 × 10^–6^ M AT-II and 2.5 ng/ml TGF-β1 were the optimal concentrations for the CRFK cells to show EMT without influencing the viability of the cells. Samples were collected at 0 h, 6 h, 12 h and 24 h. Figure 2 shows the morphology of the CRFK cells after 24 h incubation with AT-II, TGF-β1 or a combination of both. Cells incubated with TGF-β1 or a combination of TGF-β1 with AT-II showed clear EMT with more stretched fibroblastic cells, whereas the cells incubated without TGF-β1 and AT-II (control) or with AT-II showed more epithelial cells.

### qRT-PCR analyses

**Figure 3:**
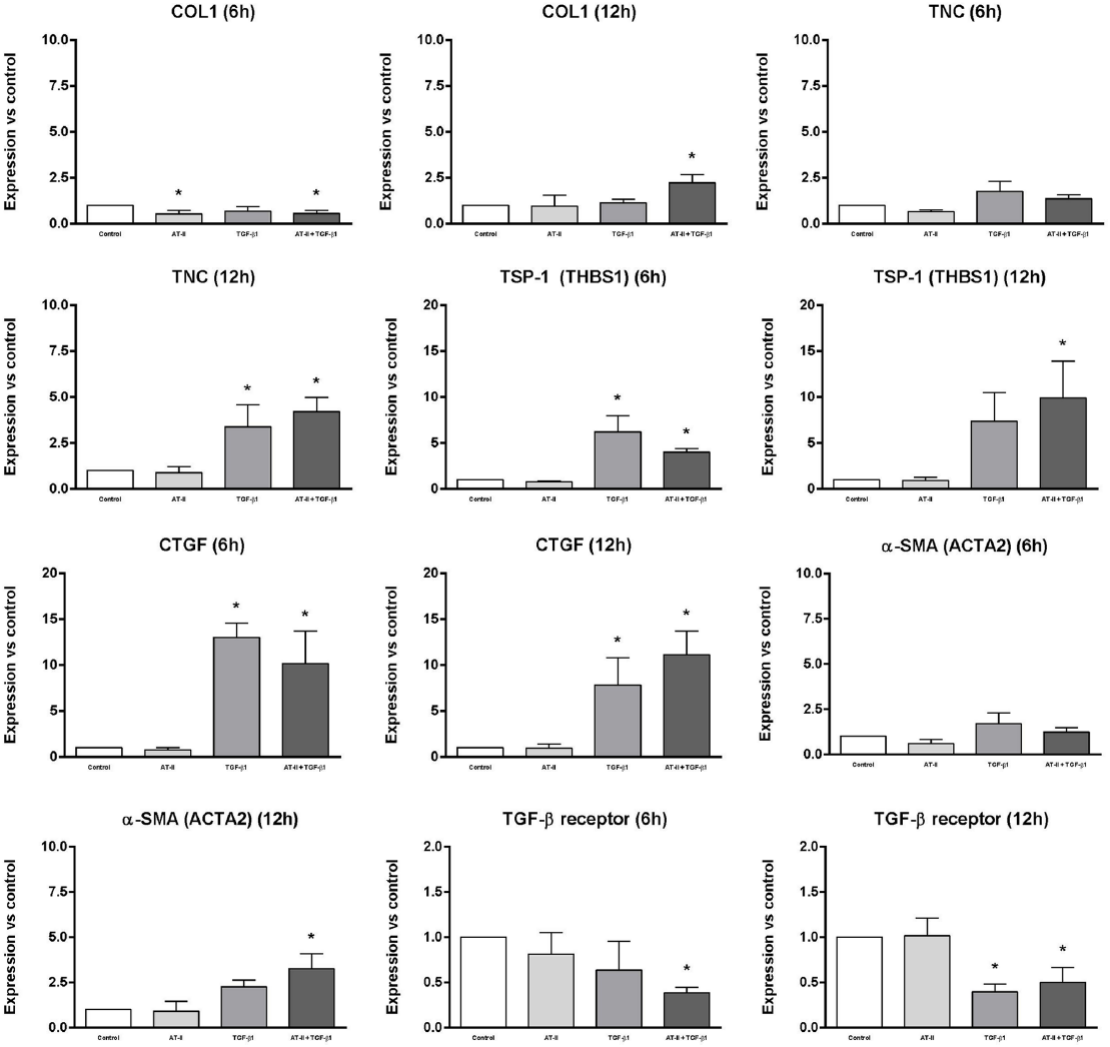
Gene expression of collagen type I (*COL1*), tenascin-C (*TNC*), trombospondin-1 (*TSP-1*), connective tissue growth factor (*CTGF*), alpha-smooth muscle actin (α*-SMA*) and transforming growth factor beta (TGF-β) receptor was determined in Crandell Rees feline kidney cells after 6 and 12 h of incubation with either 1 × 10^6^ M angiotensin II (AT-II) (light grey), 2.5 ng/ml TGF-β1 (mid-grey) or both (dark grey). Data were obtained from three independent experiments and calculated by the expression of house-keeping control gene *RPS7* as an internal standard, using the 2^–ΔΔCt^ method; data are shown as differences in gene expression relative to the control (without AT-II or TGF-β1; white). *Significantly different from control, *P* <0.05

The results of the qRT-PCR analyses are shown in Figure 3. Gene expression of *COL1*, *TNC*, *TSP-1*, *CTGF*, *α-SMA*, TGF-β receptor and AT_1_ receptor was determined in CRFK cells after 6 and 12 h of incubation with either 1 × 10^6^ M AT-II, 2.5 ng/ml TGF-β1 or both. Data were collected from three independent experiments and are shown as differences in gene expression relative to the control (without AT-II or TGF-β1) and calculated by the expression of house-keeping control gene *RPS7* as an internal standard, using the 2^–ΔΔCt^ method. Of all tested house-keeping genes,^25^ *RPS7* turned out to be the only stable house-keeping gene in CRFK cells, which is the reason that only this reference gene was used in our calculations.

As shown in Figure 3, gene expression of *COL1*, *TNC*, *TSP-1*, *CTGF* and *α-SMA* increased significantly after incubation with TGF-β1 or TGF-β1 in combination with AT-II for 12 h. An obvious rise in gene expression of *TSP-1* and *CTGF* could already be observed after 6 h incubation with TGF-β1 or TGF-β1 in combination with AT-II. Gene expression of the TGF-β receptor seemed to be downregulated after 6 h of incubation with TGF-β1 in combination with AT-II, and showed a significant decline after 12 h incubation with TGF-β1 or TGF-β1 in combination with AT-II. The addition of only AT-II did not show any significant rise nor decline in gene expression of the abovementioned genes.

### AT_1_ receptor gene expression and immunofluorescence assay

The qRT-PCR analyses did not show clear expression of the AT_1_ receptor in the CRFK cells, and, in cells incubated with AT-II, did not result in significant gene induction of the above-mentioned EMT marker genes, profibrotic mediators and matricellular proteins. To explain the lack of gene induction by the addition of AT-II alone in comparison with the obvious gene induction of AT-II in combination with TGF-β1, the presence of the AT_1_ receptor in the CRFK cells was determined in comparison with healthy feline kidney tissue. Interestingly, while the expression of the housekeeping gene *RPS7* was almost similar in the CRFK cells vs the healthy feline kidney tissue, the CRFK cells showed almost no expression of the AT_1_ receptor, whereas the feline kidney tissue showed high expression of this receptor. After immunofluorescent staining, the presence of the AT_1_ receptor was more visible in the feline kidney tissue than in the CRFK cells, where fluorescence was almost negligible (data not shown).

## Discussion

In the present study it was shown that CRFK cells incubated with TGF-β1 or a combination of TGF-β1 with AT-II showed EMT with cells having a fibroblastic phenotype, whereas cells incubated without TGF-β1 and AT-II (control) or with AT-II showed a more epithelial phenotype. Moreover, expression data demonstrated a significant increase in gene expression of not only *TSP-1* and *CTGF*, but also of *COL1*, *TNC* and *α-SMA* after incubation of CRFK cells with TGF-β1 or TGF-β1 in combination with AT-II for 12 h. Incubation with TGF-β1 or TGF-β1 in combination with AT-II for 12 h appeared to downregulate the expression of the TGF-β receptor in CRFK cells. With the change in phenotype, increased expression of the EMT marker genes, profibrotic mediators and fibrogenic proteins, it can be concluded that TGF-β1 causes renal fibrosis in CRFK cells. The expression of the AT_1_ receptor and TGF-β receptor in these cells had not been studied previously and were included in this study.

Besides the increased expression of mesenchymal cell markers such as *α-SMA*, a decline in epithelial markers such as E-cadherin could also be measured during the EMT process in human kidney cells. E-cadherin is a calcium-dependent molecule involved in cell–cell adhesion and when epithelial cells undergo EMT, the expression of E-cadherin declines. In our research we have tested several primers for the expression of E-cadherin, but none of them showed a significant expression of E-cadherin in the CRFK cells, let alone measurement of a decline in expression after EMT. It might be possible that the primers were not suitable for measuring feline E-cadherin expression, or that the CRFK cells are not totally epithelial cells to begin with. Although the CRFK cell line is ‘old’ and the question arises of whether it has retained its epithelial phenotype over multiple passages since it was first isolated and immortalised, it is the only cell line available from feline kidney tissue. Confirmation of the results of this study could therefore only be made by harvesting primary cells of feline kidney.

As AT-II contributes to renal fibrosis through gene induction, increased release and through receptor induction of TGF-β1 in humans,^13^ it was expected that this would also apply to feline kidney cells. Also, the AT-II-induced gene expression of *CTGF* and *COL1* in human kidney tubule epithelial cells would have similar effects in feline kidney epithelial cells. Because a significant rise in neither gene expression of any of the EMT marker genes nor of the profibrotic mediators or proteins was seen after the incubation of the CRFK cells with AT-II, the expression of the AT_1_ receptor was evaluated in CRFK cells and compared with healthy feline kidney tissue. The results showed almost no gene expression of the AT_1_ receptor in the CRFK cells vs a high expression in healthy feline kidney tissue, whereas expression of the housekeeping gene *RPS7* was almost similar. Immunofluorescent staining confirmed this extremely low presence of the AT_1_ receptor in the CRFK cells, which explains the lack of effect of AT-II in CRFK cells.

To our knowledge, this is the first time that the expression of the AT_1_ receptor and TGF-β receptor in CRFK cells has been evaluated. As the CRFK cell line is the only available kidney cell line originated from cats, the unexpectedly low expression of the AT_1_ receptor makes these cells less suitable for testing the effect of AT-II and subsequently the efficacy of drugs interacting with the AT_1_ receptor on renal fibrosis in cats. However, as incubation of these CRFK cells with TGF-β1 led to an induction in EMT marker genes, profibrotic mediators and matricellular proteins, it could be assumed that in feline epithelial cells the mechanism for renal fibrosis is comparable to human kidney epithelial cells. Recently published data from feline renal cortical fibroblast cultures confirm our results, as incubation of these cells with TGF-β1 also increased the expression of *COL1*, *α-SMA* and *CTGF*.^26^ Although the CRFK cells could have changed over time from more of an epithelial to a somewhat more fibroblastic phenotype, the results of the present study clearly demonstrated the formation of myofibroblasts by TGF-β1 via increased expression of *COL1*, *α-SMA* and *CTGF*.

Clinically, it was found that measuring urinary TGF-β1 levels could serve as a diagnostic tool for determining the severity of renal pathology in cats.^27–29^ As demonstrated in the present study, TGF-β1 is the main inducer of renal fibrosis by among other increasing EMT marker genes in our feline kidney cells. Drugs that modulate myofibroblast formation or the TGF-β signalling pathway could therefore offer new therapeutic strategies in the inhibition of feline renal fibrosis. The next step in unravelling the mechanism and treatment options for feline renal fibrosis would be to measure the effects of EMT-modulating or TGF-β1-inhibiting drugs in cats suffering from CKD.

## Conclusions

TGF-β1 changed the morphology of CRFK cells from a more epithelial phenotype to a more fibroblastic phenotype, and significantly induced expression of EMT marker gene *α-SMA*, profibrotic mediator *CTGF*, and matricellular proteins *COL1*, *TNC* and *TSP-1* in CRFK cells. These results further support the profibrotic effect of TGF-β1 in the kidney demonstrated by earlier (clinical) studies in cats.^26–28^ The CRFK cells showed almost no expression of the AT_1_ receptor, precluding induction of these EMT marker genes by AT-II incubation. As AT-II contributes to renal fibrosis by various mechanisms, of which one is by TGF-β1 gene induction, it can be hypothesised that AT-II would show similar results to TGF-β1 if the AT_1_ receptor was expressed more in CRFK cells.

Although the CRFK cell line is the only available kidney cell line originated from cats, this cell line seems to be not suitable for testing the effects of AT-II on feline renal fibrosis or drugs that modulate this mechanism. However, the effect of AT-II on renal fibrosis can be measured indirectly with this cell line, as TGF-β1 demonstrated EMT, indicating profibrotic effects of TGF-β1 and most probably also AT-II in feline kidney epithelial cells. With a step forward in the knowledge of the mechanism behind feline renal fibrosis, modulation of EMT or proliferation of myofibroblasts could serve as a diagnostic tool and a novel therapeutic target to inhibit renal fibrogenesis, and could possibly serve in the therapy or at least the delay of feline renal fibrosis.
